# Ultrasonic Touch Sensing System Based on Lamb Waves and Convolutional Neural Network

**DOI:** 10.3390/s20092619

**Published:** 2020-05-04

**Authors:** Cheng-Shen Chang, Yung-Chun Lee

**Affiliations:** 1 Department of Mechanical Engineering, National Cheng-Kung University, Tainan 70101, Taiwan; mr.chang100@gmail.com; 2 Center for Micro/Nano Science and Technology, National Cheng Kung University, Tainan 70101, Taiwan

**Keywords:** tactile position sensing, ultrasound, Lamb wave, convolutional neural network, steel plate, piezoelectric transducers

## Abstract

A tactile position sensing system based on the sensing of acoustic waves and analyzing with artificial intelligence is proposed. The system comprises a thin steel plate with multiple piezoelectric transducers attached to the underside, to excite and detect Lamb waves (or plate waves). A data acquisition and control system synchronizes the wave excitation and detection and records the transducer signals. When the steel plate is touched by a finger, the waveform signals are perturbed by wave absorption and diffraction effects, and the corresponding changes in the output signal waveforms are sent to a convolutional neural network (CNN) model to predict the x- and y-coordinates of the finger contact position on the sensing surface. The CNN model is trained by using the experimental waveform data collected using an artificial finger carried by a three-axis motorized stage. The trained model is then used in a series of tactile sensing experiments performed using a human finger. The experimental results show that the proposed touch sensing system has an accuracy of more than 95%, a spatial resolution of 1 × 1 cm^2^, and a response time of 60 ms.

## 1. Introduction

Touchscreens are widely used throughout daily life for such applications as mobile phones, computers, and interactive machines. The rapid development of touchscreen technology in the past few decades has fundamentally changed the way in which people interact with machines and electronic devices. According to their underlying mechanisms and designs for fulfilling tactile sensing, touchscreens can be divided into several categories, including capacitive, resistive, optical, acoustic, and so on [1]. Each type of touchscreen has its own set of pros and cons in terms of its functionality, applicability, manufacturing complexity, and cost. The present study considers the problem of turning any elastic solid panel (e.g., a metal sheet and a glass plate) into a touchscreen so that the idea of touchscreen everywhere becomes more plausible in the future.

Of the various tactile sensing technologies available nowadays, acoustic waves or ultrasounds appear to be particularly attractive solutions for the problem described above, since elastic waves can be readily excited and detected in many solid structures. Existing ultrasound touchscreens generally utilize surface acoustic waves (SAWs) for tactile sensing [1,2,3]. However, in recent years, the feasibility of using Lamb waves (or plate waves) to realize touchscreens on solid plates has gained increasing attention [4,5,6,7,8,9,10,11,12]. Depending on how the wave energy is generated, Lamb-wave-based touchscreens can be classified as either passive or active. In touchscreens of the former type, the Lamb waves are generated via the application of pressure to the sensing surface by the human finger and are detected by acoustic sensors strategically positioned on the plate [4,5,6]. However, while such devices have the advantages of structural and operational simplicity, they cannot operate for a still finger touch on the plate. In active Lamb-wave touchscreens [7,8,9,10,11,12], the Lamb waves are typically generated and detected by piezoelectric transducers, which are attached to the plate and constantly excited by an electrical signal [13,14]. Both anti-symmetrical modes [7,8,9] and symmetrical modes [10,11,12] of Lamb waves had been applied. For anti-symmetrical modes, the waves were excited by attaching piezoelectric transducers on the planar surfaces of the plate [7,8,9,13,14]. For symmetrical modes, PZT transducers were directly attached to the side surface of the plate [10,11,12]. The application of finger pressure to the plate perturbs the wave signal and induces a corresponding change in the outputs of the detection piezoelectric transducers from which the occurrence and position of the tactile event can then be derived.

The most challenging problem in designing any active Lamb-wave touchscreen is that of predicting the tactile position of the finger on the plate based on the received wave signals. A number of localization algorithms have been proposed before. Examples are amplitude disturbed diffraction pattern (ADDP) [7,8], contact impedance mapping method [9], and projection method in the vector space of collected training data [10,11,12]. One common feature in these methods is that a huge number of testing signal data for touching at different locations were first collected, either experimentally or numerically, and then stored in a computer, to form a database. For a truth touching event, the corresponding signal data is then processed, along with the database, through a certain algorithm, to identify the touching position. Due to the complexity of various active ultrasound touchscreens, it is very difficult to justify or evaluate the reliability and robustness of these pattern-recognition algorithms.

The present study developed an active Lamb-wave touchscreen based on a steel plate and a number of piezoelectric transducers. The transducers are mounted around the perimeter of the underside of the plate for Lamb-wave generating and detecting purposes, and the area surrounded between these transducers on the reverse (i.e., upper) surface of the plate becomes the tactile sensing area. When a fingertip is applied to this sensing area, the wave signals traveling through the plate are perturbed, and the resulting changes in the output signals of the receiving transducers are then used to determine the finger contact position. Artificial Intelligence (AI) technologies have advanced at an incredible speed in recent decades and are now used for solving complicated problems in many different areas. Recent studies in the field of structural health monitoring (SHM) have shown that AI has significant potential for dealing with the complex phenomena associated with wave propagation through solid structures, even under complicated circumstances [15,16,17,18]. Among the various deep-learning models available, convolutional neural network (CNN) models have proven to be particularly effective in solving pattern-recognition and signature-identification problems [19,20]. Hence, to deal with the challenging issue of localization algorithm in tactile position sensing, the waveform signals produced by the detection piezoelectric transducers of the touchscreen proposed in the present study are processed by CNN. To the best of our knowledge, this is the first time the use of the deep machine learning algorithm of CNN to solve the ultrasonic tactile position sensing problems has been introduced.

## 2. Construction of Touchscreen System

**Figure 1:**
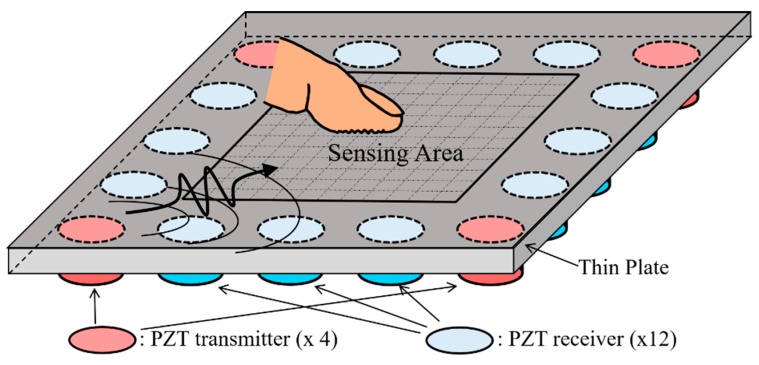
Schematic illustration of Lamb-wave-based ultrasonic touchscreen based on thin plate as touch panel and disk-shaped piezo-transducers as wave transmitters (four in present diagram) and receivers (twelve in present diagram). Tactile position sensing is conducted on upper surface of plate, while piezo-transducers are attached on under surface of plate.

Figure 1 presents a schematic illustration showing the basic concept of the proposed Lamb-wave-based ultrasonic touchscreen. As shown, the device consists of a thin solid plate serving as the touchscreen panel and 16 piezoelectric transducers uniformly deployed around the outer perimeter of the lower surface of the plate. Four of the transducers (i.e., the corner transducers) serve as wave transmitters, which, after being electrically excited, launch acoustic waves into the plate, while the remaining transducers serve as wave receivers, which output electrical signals when they receive acoustic waves from the plate. The area enclosed by the piezo-transducers on the upper surface of the plate represents the sensing area of the touchscreen. In theory, both surfaces of the plate could be used as the sensing surface. However, in the present study, the opposite side of the transducer-mounting surface is deliberately chosen as the tactile sensing surface, since this minimizes interference with the finger contact process and also offers better protection to the piezo-transducers and their electrical wirings. To facilitate tactile position sensing, acoustic waves are continuously excited in the plate by voltage signals, and the received wave signals are constantly monitored by a personal computer (PC). Furthermore, the signals received by the PC are interfaced continuously to an artificial intelligence (AI) model implemented on the PC, to determine the occurrence of finger contact and, if so, the x- and y-coordinates of the touched position.

**Figure 2:**
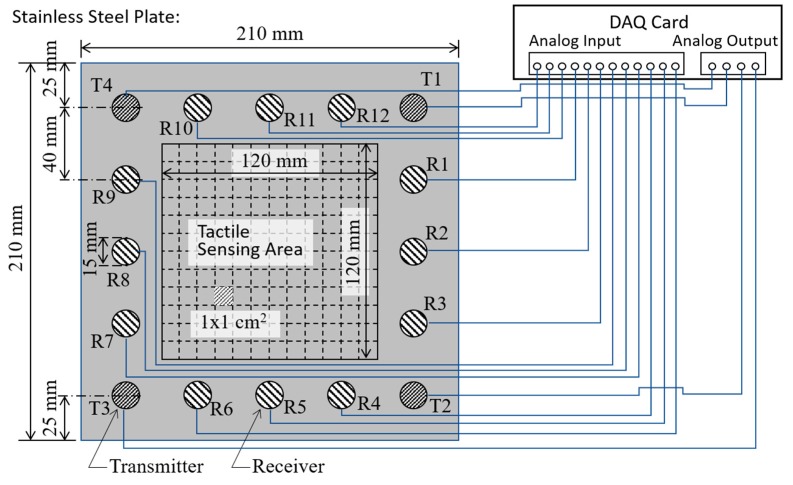
Schematic illustration showing dimensions of stainless-steel plate and locations of 16 disk-shaped piezo-transducers, together with associated wiring between transmitting/receiving piezo-transducers and analog output/input ports of DAQ card. Note that T1~T4 are transmitting piezo-transducers, while R1~R12 are receiving piezo-transducers.

In the present study, the thin plate used as the touchscreen had the form of a 0.8 mm thick stainless steel (SS 304) plate with a size of 210 mm × 210 mm. The disk-shaped piezo-transducers were fabricated of PZT-5A piezo-ceramic material (Eleceram Inc., Taoyuan, Taiwan) and had a diameter and thickness of 15 and 0.6 mm, respectively. The disks were coated with inverted electrodes, such that they could be firmly epoxy-glued to the steel plate and easily connected to the electrical wiring harness. As shown in Figure 2, the 16 piezoelectric transducers were symmetrically deployed at a distance of 25 mm from the edge of the plate and were positioned such that they formed a square shape with a center-to-center distance of 40 mm between them. Among the 16 piezoelectric transducers, four of them (T1~T4) located at the corners of the steel plate were chosen as wave transmitters, while the other twelve piezo-transducers (R1~R12) were designated as wave receivers. During operation, the PZT transmitters were electronically excited, and the resulting acoustic waves propagated along all directions of the plate were received by the 12 receivers. As shown in Figure 2, the tactile sensing area enclosed by the 16 transducers had a size of 120 mm × 120 mm and was uniformly partitioned into an array of 12 × 12 square blocks, each with a size of 1 × 1 cm^2^. In practice, each square block represents a possible tactile sensing position, and hence the ultrasonic tactile position sensing system provided a total of 144 possible position outputs with a spatial resolution of 1 × 1 cm^2^. The ultrasound touchscreen is now functioning as a keypad with an array of 12 × 12 keys.

To facilitate the excitation and detection of the acoustic waves, the piezo-transducers were electrically connected to a PC-based data acquisition card (DAQ card, PXIe-6358, National Instrument, Austin, TX, USA). As shown in Figure 2, the DAQ card was equipped with 16 analog input channels and 4 analog output channels. Among the input channels, twelve of them were connected to the receiving piezo-transducers (R1~R12), to acquire the received acoustic waves with a maximum data acquisition rate of 1.25 MHz and a voltage resolution of 16 bits. Meanwhile, the four output channels were connected to the four transmitting piezo-transducers (T1~T4), to apply arbitrary excitation waveforms. The generation of the output voltage signals used to perform wave excitation was synchronized with the digitization of the input waveform signals by the host PC (PXIe-8861, National Instrument, Austin, TX, USA), and the resulting wave signals were then processed by the PC and interfaced to the AI model.

**Figure 3:**
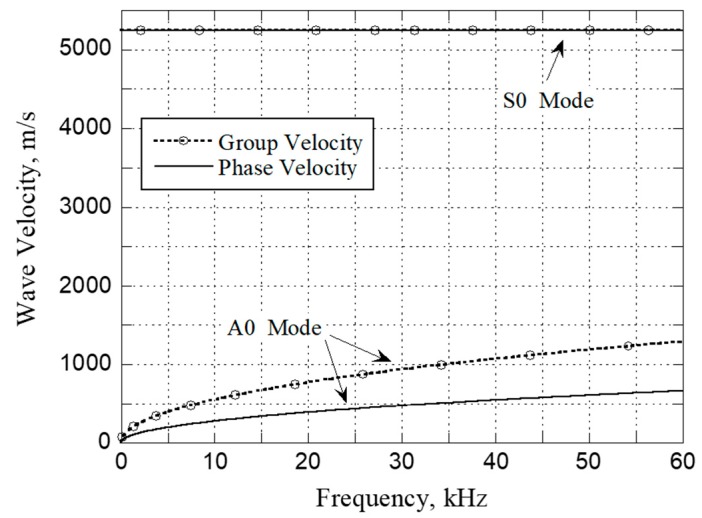
Dispersion curves of A0 and S0 modes of Lamb waves in a 0.8 mm thick stainless steel (SS 304) plate.

For the 0.8 mm thick stainless-steel plate used in the present study, the longitudinal and shear wave velocities were found to be 5760 and 3128 m/s, respectively, as measured using conventional contact ultrasound transducers. The corresponding dispersion curves of the Lamb waves are shown in Figure 3. Since the plate is relatively thin, only the fundamental symmetric (S0) and anti-symmetric (A0) modes of the Lamb waves can propagate in the lower frequency range, as shown in Figure 3. Previous studies have shown that, for any pair of disk-shaped piezo-transducers mounted on a plate, the most effective excitation and detection of acoustic waves in the plate is achieved by using A0 mode Lamb waves [13,14]. For the 15 mm in diameter piezo-transducers and 0.8 mm thick stainless-steel plate used in the present study, the optimal operating frequency is essentially that which achieves the best trade-off between the enhancing wave signals and the reducing acoustic noise. Based on a series of experimental trials, the operating frequency was thus set as 35 kHz, for which the group velocities of the A0 and S0 modes were 1002 and 5246 m/s, respectively. In the tactile-sensing experiments, a five-cycle 35 kHz voltage signal modulated by a Hamming window with a maximum amplitude of 10 V was applied to the transmitting piezo-transducers, and the resulting acoustic waves propagated through the steel plate in all directions and were continuously reflected between the plate boundaries. When the Lamb waves arrived at a receiving piezo-transducer, a voltage signal was generated by the transducer, and the resulting signal waveform was digitized by the analog input channel of the DAQ card and recorded by the PC.

**Figure 4:**
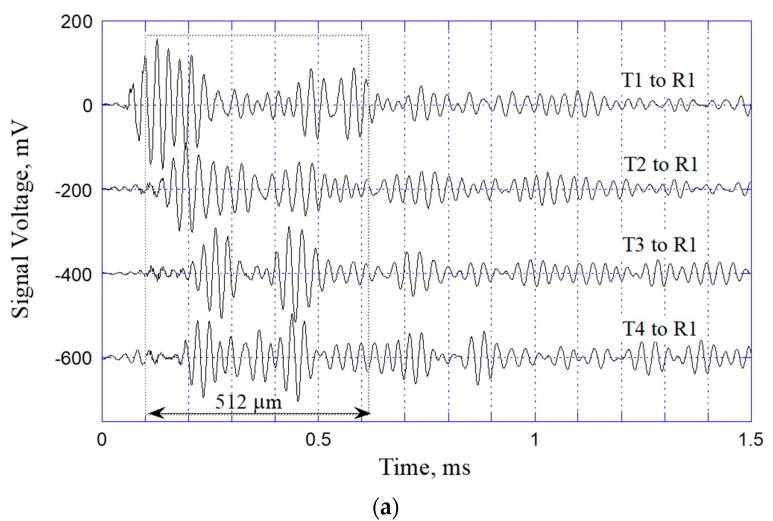

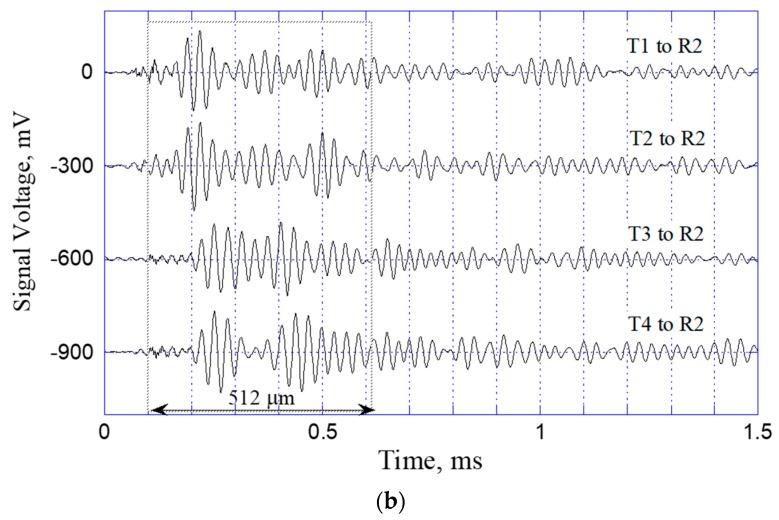
Signal waveforms of Lamb waves launched by T1, T2, T3, and T4, respectively, and received at transducers (**a**) R1 and (**b**) R2.

Figure 4a,b show the typical signal waveforms detected at R1 and R2, respectively (see Figure 2). Referring to the third waveform (T3 to R1) in Figure 4a, for illustration purposes, the center-to-center distance between T3 and R1 is equal to approximately 200 mm, and the travel time of the A0 mode Lamb wave with a group velocity of 1002 m/s is thus equal theoretically to 0.2 ms. A close inspection of the waveform confirms that the first arrival of the A0 mode Lamb wave indeed occurs after 0.2 ms. Moreover, it is apparent that the A0 mode of the Lamb wave dominates the wave signal. In other words, while the S0 mode Lamb wave, which has a much higher group velocity of 5246 m/s, can also be detected in front of the A0 signal, it has a much weaker signal amplitude. Following the first arrivals of the S0 and A0 modes of the Lamb wave traveling directly from T3 to R1, the detected waveform contains multiple peaks corresponding to the arrival of wave reflections and mode-conversions at the plate boundaries. Similar tendencies are observed in all of the other signal waveforms detected at R1 and in the waveforms detected at R2 (see Figure 4b). Due to the positional symmetry of R2 with respect to T1/T2 and T3/T4 on the plate, similarities are also observed between the first two waveforms in Figure 4b (i.e., T1 to R2 and T2 to R2) and the last two waveforms (i.e., T3 to R2 and T4 to R2). As discussed later, in Section 3, the positional symmetry of the transmitting/receiving transducers plays a key role in obtaining a reliable AI model for tactile position sensing.

The waveforms shown in Figure 4a,b are obtained when the steel plate is not touched. As such, they serve as a useful source of reference for evaluating the occurrence (or otherwise) of fingertip contact on the sensing surface. In particular, the difference between the wave signals received before and after finger contact, respectively, provide the means to detect both the occurrence of a finger contact event and (if so) the position of this contact event on the sensing surface. However, analyzing the waveforms and their changes analytically is extremely challenging, if not impossible. Furthermore, the analytic analysis must take the boundary conditions of the plate into account, and this further complicates the analysis process. Thus, in the present study, the detected signal waveform data are input to an AI model, to carry out tactile position sensing (see Section 3).

**Figure 5:**
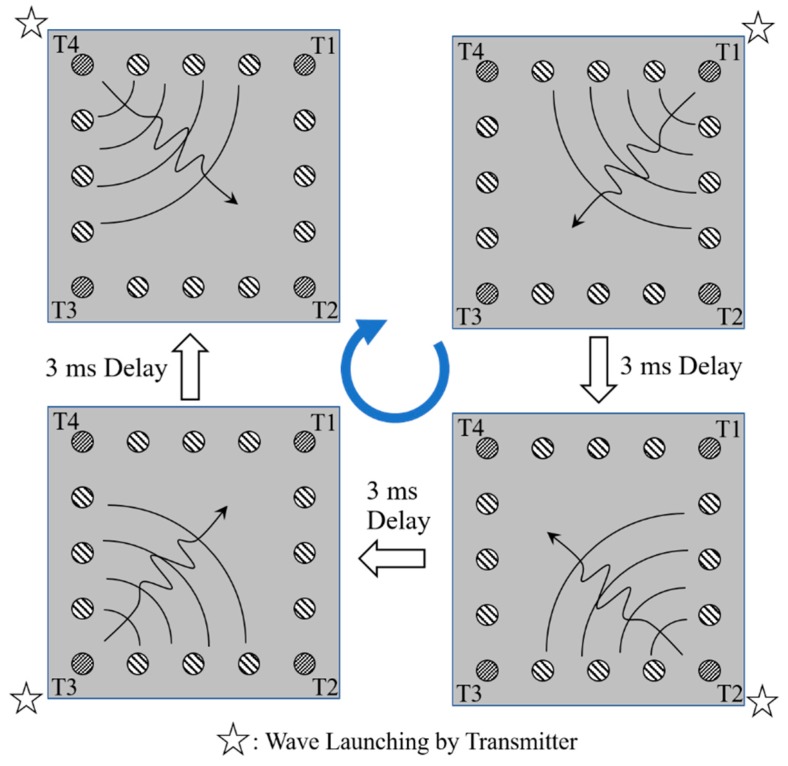
Complete cycle for launching acoustic waves into steel plate by four transmitters (T1~T4) with 3 ms time delay between them. The 12 receivers are synchronized to continuously receive wave signals over the 12 ms cycle.

Experimentally, the proposed PZT/steel-plate tactile sensing system is operated by sequentially exciting the four transmitting transducers in turn, as shown in Figure 5. Assume that T1 is excited first and launches acoustic waves into the steel plate accordingly. The 12 receivers record the received wave signals simultaneously and interface them to the PC through the DAQ card (see Figure 4a,b, for example). The acoustic waves bounce back and forth between the plate boundaries and continue to be detected by the receivers. However, the intensity of the reflected waveforms gradually reduces as a result of energy dissipation, and hence, after a certain period of time, the second transmitter (T2) is excited and launches its own acoustic waves into the plate. Based on an observation of the decaying waveform signals, the optimal time delay between successive excitation voltages was determined to be 3 ms. Consequently, the total data acquisition time for a single tactile sensing event was equal to 12 ms. Each of the 12 receivers thus produced a synchronized 12 ms long signal waveform containing four equal-length segments of 3 ms duration, corresponding to the waveforms excited sequentially by T1 to T4, respectively. Data acquisition was carried out with a sampling frequency of 1 MHz, and thus a total of 12,000 data points was acquired for each 12 ms waveform. However, the results obtained from a series of preliminary tactile sensing tests revealed that the most significant changes in the received waveforms typically occurred between 0.1 and 0.6 ms after the acoustic waves were launched by the transmitters. Thus, as shown by the dashed rectangles in Figure 4, data collection was performed only over a period of 512 ms (or 512 data points), starting after 100 ms (or 100 data points) for each waveform. For each receiver, the four segments of data points corresponding to the acoustic waves launched by the different transmitters were edited into a single waveform consisting of 2048 (4 × 512) data points. Finally, the 12 × 2048 data points collected from all 12 receivers were interfaced to the deep-learning model installed on the PC, to perform tactile position sensing.

## 3. Convolutional Neural Network (CNN) Model and Experimental Results 

As described above, for each single touch event, a total of 12 waveforms, each with a length of 2048 (4 × 512) data points in the time domain, are collected simultaneously by the PC. The acquired waveforms are subtracted from their corresponding reference waveforms before the plate is touched, and the resulting difference waveforms are compiled to create an “image” with a size of 12 × 2048 pixels. The input data image is then provided to an AI model, which examines the image features and determines the position of the tactile event on the sensing surface accordingly.

Convolution neural networks (CNNs) have undergone rapid development in the past few decades and have found widespread use for many different image-recognition applications. CNNs based on ultrasound or acoustic waves have also been successfully applied for structural health monitoring [15,16,17,18] and non-destructive evaluation [21,22,23]. However, thus far, the literature contains scant information on the use of CNNs for tactile sensing on ultrasound touchscreens. Generally speaking, two different types of CNN model exist, namely *unsupervised* models, which are used mainly for the classification of objects, and *supervised* models, which are designed to perform the recognition of particular targets through the use of a supervised training process. Intuitively, the problem considered in the present study, namely that of detecting a tactile event and (if so) establishing its position on the sensing service, is best solved by using a CNN model of the latter type.

**Figure 6:**
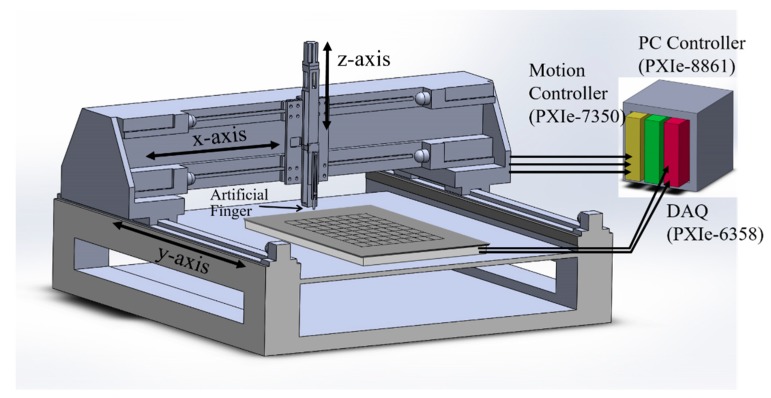
Collection of training data for Lamb-wave touchscreen using artificial gelatin finger carried by three-axis (x–y–z) automatic stage integrated with PC-based data acquisition system.

**Figure 7:**
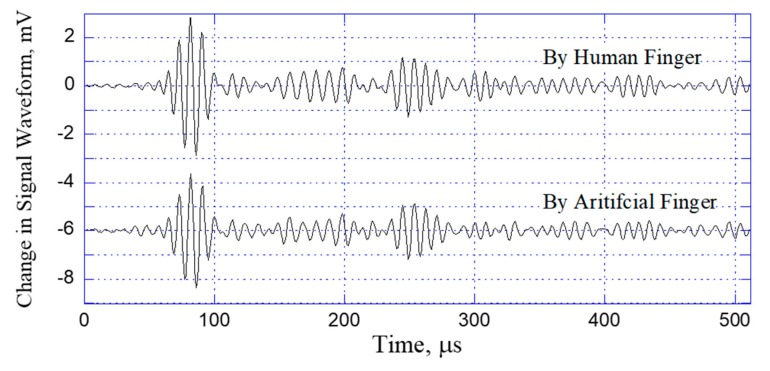
Comparison of changes induced in received acoustic waveforms by human finger and gelatin artificial finger.

In developing any supervised CNN model, one of the most critical challenges is that of obtaining sufficient and reliable training data to ensure the accuracy and robustness of the trained model. As shown in Figure 6, the training data for the present CNN model were acquired by using a three-axis (x–y–z) automated stage integrated with the signal waveform-data acquisition system under the control of the PC. During the collection procedure, an artificial finger was mounted vertically in the z-stage and was driven by the controller such that it touched the steel plate at each sensing position on the plate surface. For each touch event, the acquired waveform signals were automatically saved to the PC, together with the corresponding x- and y-coordinates of the touched position. To ensure the robustness of the training process, the changes in the received acoustic waveforms induced by the touch of the artificial finger must be very similar to those induced by a true human finger. Following a series of experimental trials performed using artificial fingers fabricated of different materials, a 20 mm long cylinder made of gelatin with a diameter of 10 mm, and a loading force of 0.2 N was found to provide the best fit to the waveforms produced by a human finger. The artificial finger is held by a spring which is firmly connected to the z-axis auto-stage. By controlling the vertical displacement along z-axis, we can maintain the constant 0.2 N contact force. Figure 7 compares the changes induced in the signal waveforms by the human finger and the gelatin finger, respectively. (Note that, as described above, each signal waveform was obtained by subtracting the received waveform from the originally untouched waveform and consisted of only the 512 data points within the time period of 0.1 to 0.612 ms after wave excitation.) The results confirm that the two signal waveforms are almost identical, and hence the validity of the gelatin finger for training data acquisition is confirmed. 

**Figure 8:**
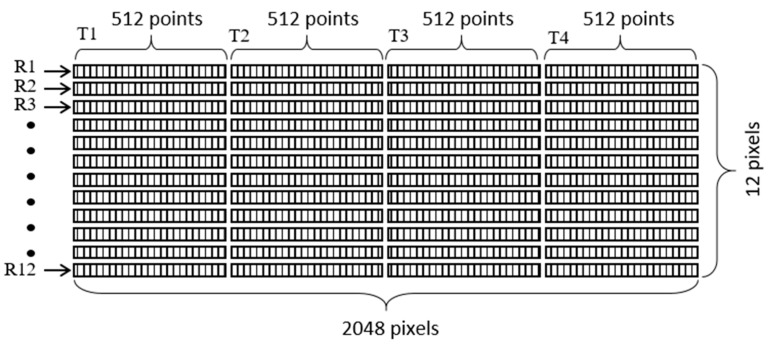
Construction of input image to CNN model for acoustic tactile position sensing. Image has dimensions of 12 × 2048 pixels corresponding to finger-touch-induced changes in acoustic waveforms received by 12 receivers (R1 to R12), following sequential generation of acoustic waves by four transmitters (T1 to T4).

As shown in Figure 2, the 12 × 12 cm^2^ sensing area of the steel plate contains a total of 144 (12 × 12) touch positions, each with a size of 1 × 1 cm^2^. During the training data-collection process, the gelatin finger was pressed into intimate contact with each of these touch positions, one by one. The PC-controlled wave excitation and waveform acquisition system collected the resulting changes in the received acoustic waveforms and stored them to the PC. As shown in Figure 5, for each finger contact, the four transmitting piezo-transducers were excited in sequence, and the 12 receiving piezo-transducers collected the waveform data simultaneously. Finally, after waveform subtraction and editing of the four segments of 512 data points in each receiving channel, the obtained data were compiled into a grayscale image with a size of 12 × 2048 pixels, as shown in Figure 8. (Note that the grayscale levels in the image indicate the waveform amplitude after normalization.)

**Figure 9:**
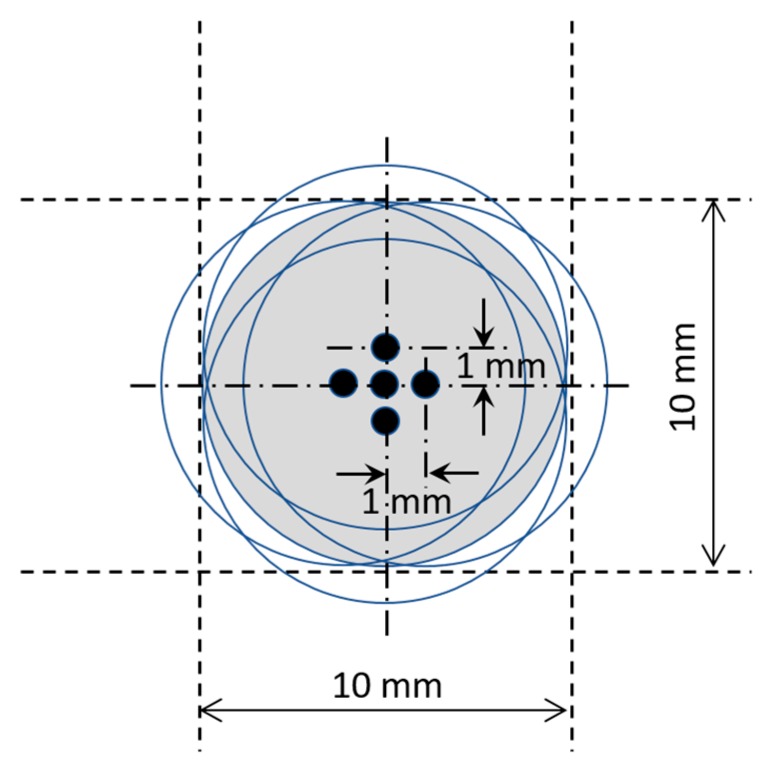
Multiple artificial-finger touch positions at each sensing position on sensing area.

In real-world applications, the accuracy of a real human-finger touch on a specific touch position on the sensing surface will never be as accurate as that of the artificial finger controlled by the stage. Accordingly, training data were collected not only for artificial finger contacts at the center of each sensing position, but also at the four neighboring points located at a distance of 1 mm from the center position in every direction (see Figure 9). In other words, for each touch position in the sensing area of the steel plate, five sets of training data were acquired. To improve the signal/noise ratio, the data acquisition process was repeated 100 times at each sensing position. Consequently, a total of 144 × 5 × 100 images was collected for the supervised training process, with each image having a size of 12 × 2048 pixels.

**Figure 10:**
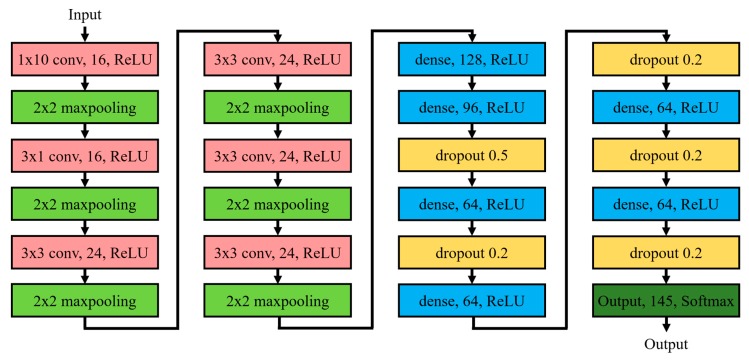
Architecture of CNN model for Lamb-wave touchscreen position sensing.

In developing the AI model for the Lamb-wave-based ultrasound touchscreen, this study commenced by constructing a simple CNN model architecture consisting of an input layer, several convolution layers and fully-connected dense layers, and an output layer. In practice, determining the optimal CNN architecture and related parameter settings is somewhat arbitrary and depends heavily on both earlier studies on CNNs for pattern recognition and the nature of the training data collected by using the process described above. After a number of numerical trials, the optimal CNN architecture for the considered tactile positioning sensing problem was found to be that shown in Figure 10, namely an input layer, six convolution layers, six fully-connected dense layers, and an output layer. The input image (a gray-scaled image with a size of 12 × 2048 pixels) was first processed by the six convolution layers, to extract the key features related to tactile position sensing. The kernel filters used in the six convolution layers are shown in Table 1, together with the associated activation and pooling methods. After the convolution layers, the data were fully connected with six dense layers with different numbers of neurons, ranging from 128 to 64. Finally, the data were passed to a fully connected output layer with 145 outputs, corresponding to one Non-Touch output and 144 touching position outputs, respectively. Table 2 summarizes the parameters and operations (activation and dropout) used in the fully connected layers and output layer.

Once the architecture of the CNN model was established, the training data were used to train the model and optimize its internal parameters. The model was implemented in a C++ environment in the PC controller, using Python and the Tensorflow library, both of which have been widely used in AI applications. To confirm the validity of the established CNN model, 70% of the collected training data were used to train the CNN model under supervision, while the remaining 30% were used to test the trained model and determine its accuracy. Once the CNN model can accurately predict correct touch positions to the input data, the whole tactile position sensing system is then tested in situ and in real time by human-finger touches.

**Figure 11:**
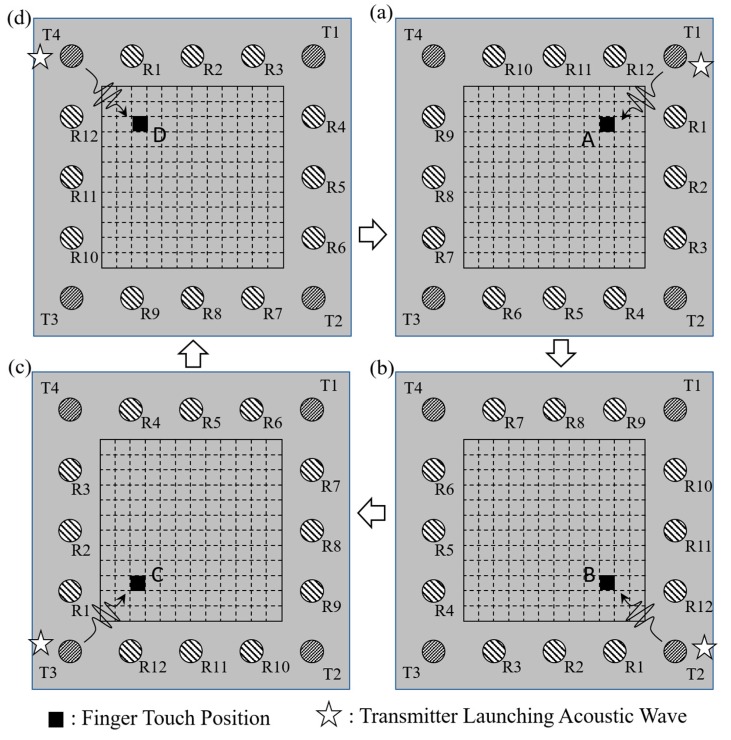
Rotating numbering system for 12 receivers (R1 to R12), based on launching transmitter at (**a**) T1, (**b**) T2, (**c**) T3, and (**d**) T4.

In training the CNN model, it was found that the formation of the input image (see Figure 8), along with the assignments of T1 to T4 and R1 to R12 shown in Figure 2, plays a dominant role. In particular, it was found that the use of a “fixed” numbering policy for the transmitters and receivers when constructing the input image resulted in a suboptimal training performance. Consequently, the training process was repeated by using a “rotating” numbering policy for the transmitters and receivers instead. Specifically, the numbering of the 12 receivers was updated following each excitation event, depending on the particular transmitter launching acoustic waves into the plate. More particularly, the receiver closest to the transmitter was numbered as R1, and the remaining receivers were then numbered sequentially from 2 to 12, in the clockwise direction, as shown in Figure 11. The acquired waveform data were then compiled into an input image with the same format as that given in Figure 8. The results showed that the use of the “rotating” numbering policy yielded an effective improvement in the accuracy and stability of the CNN model following model training.

**Figure 12:**
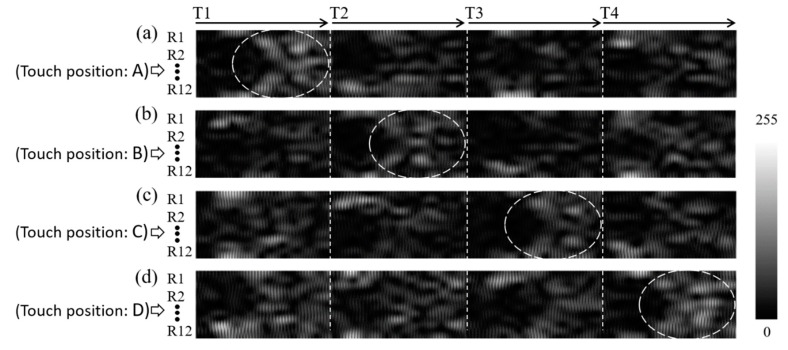
Experimentally obtained waveform data images for touch positions (**a**) A, (**b**) B, (**c**) C, and (**d**) D as shown in Figure 11, respectively.

To better understand the effectiveness of the rotating numbering system in improving the performance of the training process, consider the four touching events located at positions A, B, C, and D, in Figure 11a–d, respectively. Note that the touching positions are rotationally symmetrical to one another. Thus, if the rotating system is adopted for numbering the receivers, the data images constructed for the four touching events also bear symmetry information. Figure 12a–d shows the four data images obtained in the training data-collection process when touching the sensing surface at positions A, B, C, and D, respectively. As shown, the similar features in the four images obtained at the different contact positions move progressively from T1 to T4, due to the symmetry between the touch position and the transmitter/receiver configuration. In other words, the CNN model has a better chance of recognizing the feature similarity among the four touching events shown in Figure 11 and classifying them into a single category. The same condition applies for all 144 touch positions on the sensing surface, and hence the CNN model can classify the 144 possible outputs into just 36 (144/4) subgroups, thereby significantly improving the accuracy and stability of the CNN model. Referring to the four images shown in Figure 12 once again, it is seen that a certain degree of symmetry also exists with respect to the horizontal central line of the image. This observation is reasonable since the considered touch positions, A, B, C, and D, are symmetrical with respect to launching transmitters T1, T2, T3, and T4, respectively, when using the rotating numbering system. This symmetry feature is also beneficial to the CNN model in distinguishing the touch positions and grouping them into different categories for pattern recognition purposes.

**Figure 13:**
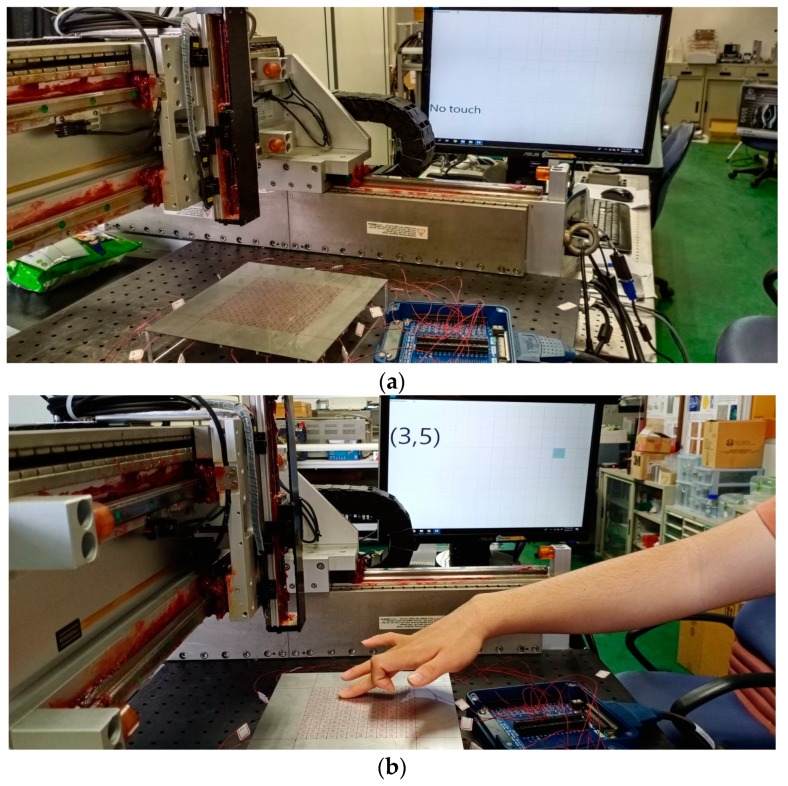
Photos of human-finger testing on the Lamb-wave ultrasound touchscreen when there is (**a**) no touch and (**b**) finger touch at the position coordinates of (3, 5) on the steel plate.

The practical feasibility of the Lamb-wave touchscreen and CNN model was evaluated by performing a series of human-finger touch tests, in which 10 volunteers were asked to touch the sensing area of the steel plate with their finger, at 100 different positions randomly selected. Each volunteer recorded his/her 100 touched positions and checked with his/her corresponding output coordinates from the ultrasound measurement system and the CNN model, as shown in Figure 13a,b. The touchscreen sensing system was operated actively and constantly throughout the testing process. As described above, the entire sensing cycle (i.e., waveform launching and data acquisition) required 12 ms to complete. Moreover, an additional period was also required to perform data processing and CNN model calculation. The total response time for identifying each touch event and updating the touch position coordinates was found to be around 60 ms, corresponding to a refresh rate of approximately 17 Hz. Based on the results obtained from a thousand human-finger tests from the 10 volunteers, the accuracy of the Lamb-wave touchscreen and trained CNN model was found to be more than 95%. This performance is reasonable, since slight differences inevitably exist between the contact behaviors of the human fingers in touching the sensing surface and the more predictable behavior of the gelatin finger driven by the x–y–z stage.

## 4. Conclusions

This study proposed a new type of tactile position sensing system based on the propagation of Lamb waves in a plate. In the proposed system, the Lamb waves are excited and detected by an array of 16 piezo-transducers mounted around the perimeter of the underside of the plate, where four of these transducers (those mounted at the corners) serve as wave transmitters for launching acoustic waves into the plate, while the other 12 transducers serve as wave receivers for detecting the Lamb wave signals. The surface area encompassed by these piezo-transducers on the upper side of the plate then becomes the tactile position sensing area. The application of fingertip pressure to the sensing area results in a change in the acoustic waveform signals detected by the receiving piezo-transducers, from which the position of the contact event is subsequently determined by using a CNN model trained with the data acquired by using a position-controlled artificial finger. The experimental results obtained by using a human finger have shown that the proposed sensing system achieves a positioning accuracy of more than 95% and a response time of 60 ms. The proposed method bears certain similarities to the guided-waves tomography method [24,25,26] used in structural health monitoring and non-destructive evaluation. However, wave diffraction phenomena induced by a finger touch at a plate’s surfaces is much less that that by internal structures defects. Moreover, the requirement for fast response time is much more stringent in the proposed ultrasound touchscreen system.

The success of the proposed Lamb-wave touchscreen stems from several key factors. First of all, the symmetry of the piezo-transducer deployment on the plate allows the CNN model to classify the input image data into a relatively small number (36) of distinct categories, and it therefore improves the accuracy and stability of the position-sensing results. The use of a rotating numbering system for labeling the individual wave receivers when constructing the waveform image data for the CNN model is also beneficial in preserving the symmetrical nature of the input data and improving the ability of the model to distinguish the touch position as a result. Secondly, for practical reasons, the data needed to train the CNN model can only be collected by a machine-based system with an artificial finger. However, the similarity between the signals generated by the artificial finger and those produced by a human finger is an important concern. In the present study, this problem has been addressed by using a cylindrical artificial finger made of gelatin, with a diameter of 10 mm. The results have confirmed that the signal waveforms produced by the gelatin finger are in extremely good agreement with those produced by a human finger. Thus, the validity of the waveform data collected by the artificial finger for training purposes is confirmed. However, despite the validity of the gelatin finger as a data-collection tool, positional differences inevitably exist between the accuracy of the gelatin finger and that of the human finger when touching the specified sensing position on the touchscreen surface. To resolve this problem, training data were collected not only at the center of each sensing position on the touchscreen surface, but also at four neighboring points located at a distance of 1 mm from the center position. In other words, a total of five sets of training data were captured for each sensing position. The experimental results showed that the augmented training dataset thus obtained significantly improved the robustness of the trained CNN model.

The significance of the sensing strategy proposed in this study lies in the facility it provides to transform a regular solid plate into a touchscreen by simply mounting piezo-transducers around its edges. Notably, the proposed method can be easily applied to any material, e.g., glass, metal, ceramic, plastic, and so forth, without altering its material properties or original functions, provided that the PZT transducers can be mounted and the Lamb waves excited and detected. This paves the way for the development of a wide range of potential applications of touchscreens for tactile position sensing in daily life. For example, a glass window panel can be transformed into a tactile position sensor without interfering with its lighting function, or the metal panels used in typical household appliances such as refrigerators, washing machines, kitchen cabinets, and so on, can be used to realize human–machine interaction through touch and position sensing. To support these goals, future studies may usefully consider such problems as further reducing the response time, extending the sensing strategy to the case of multiple simultaneous touching events, and miniaturizing the data acquisition system and CNN model into a simplified and integrated electronic unit.

## Figures and Tables

**Table 1:** Parameters and operations used in convolutional layers.

	Filter	Activation	Pooling
Convolutional layer 1	16 (1,10)	ReLU	Max Pooling (2,2)
Convolutional layer 2	16 (3,1)	ReLU	Max Pooling (2,2)
Convolutional layer 3	24 (3,3)	ReLU	Max Pooling (2,2)
Convolutional layer 4	24 (3,3)	ReLU	Max Pooling (2,2)
Convolutional layer 5	24 (3,3)	ReLU	Max Pooling (2,2)
Convolutional layer 6	24 (3,3)	ReLU	Max Pooling (2,2)

**Table 2:** Parameters and operations used in dense layers and output layer.

	Neuron Unit	Activation	Dropout
Dense layer 1	128	ReLU	
Dense layer 2	96	ReLU	0.5
Dense layer 3	64	ReLU	0.2
Dense layer 4	64	ReLU	0.2
Dense layer 5	64	ReLU	0.2
Dense layer 6	64	ReLU	0.2
Output layer	145	Softmax	
